# Comparative physiological and transcriptome analyzes reveal the function of exogenous dopamine in improving the tolerance to salt stress in *Vitis vinifera* L

**DOI:** 10.3389/fpls.2026.1823723

**Published:** 2026-05-20

**Authors:** Ziguo Zhu, Feifei Han, Yusen Wu, Li Liu, Yan Han, Xiujie Li, Guirong Li, Bo Li

**Affiliations:** 1 Shandong Academy of Grape, Shandong Academy of Agricultural Science, Jinan, China; 2 College of Horticulture and Landscape Architecture, Henan Institute of Science and Technology, Xinxiang, China

**Keywords:** abiotic stress, dopamine, grapevine, salt tolerance, transcriptomics

## Abstract

**Introduction:**

Dopamine (DA) is a crucial bioactive substance. It primarily functions as a neurotransmitter in animal nervous systems, where it regulates emotion and motor control. However, its physiological roles in plants, particularly its regulatory mechanisms in abiotic stress responses remain unclear. This study systematically reveals, for the first time, the regulatory effects of exogenous dopamine on salt stress tolerance in grapevines.

**Method:**

*Vitis vinifera* ‘Summer Black’ was selected as the experimental material, and six treatments were established, including one salt concentration (200 mmol L^-1^ (mM) NaCl) and five dopamine concentrations (0.2, 0.4, 0.6, 0.8, 1.0 mM) under salt stress. Measurements of physiological indicators—such as plant phenotype, pigment content, photosynthetic performance, reactive oxygen species, and antioxidant enzyme activity—were conducted alongside transcriptome analysis.

**Results:**

Growth analyses showed that 0.4 mM DA treatment significantly alleviated growth inhibition induced by salinity (200 mM NaCl), as evidenced by plant height, root fresh weight and root dry weight being maintained at 94.99%, 88.07% and 87.37% of the control group (CK) levels, respectively. Physiological analyses demonstrated that, compared with salt treatment alone, DA treatment reduced relative electrical conductivity by 37.43%, decreased reactive oxygen species (H_2_O_2_ and O_2_^−^) levels by 23.6%, lowered malondialdehyde (MDA) content by 53.4%, and effectively regulated ion homeostasis, reducing leaf Na^+^ content by 48.2% and Na^+^/K^+^ ratio by 56.0%. Furthermore, DA treatment substantially enhanced photosynthetic efficiency (38.2% increase in Pn) and antioxidant enzyme activities (42.9 – 187.3% increases in SOD, POD, CAT, GPX and GR). Transcriptome analysis revealed that DA-regulated differentially expressed genes (DEGs) were predominantly enriched in glutathione metabolism and plant hormone signal transduction pathways, with particularly notable expression changes in glutathione-S-transferases (GSTs) and auxin signaling-related genes.

**Discussion:**

Dopamine alleviates salt stress in grape by improving photosynthetic efficiency, antioxidant enzyme activities and maintaining ion balance, meanwhile glutathione metabolism and hormone signaling may be involved in dopamine-mediate salt tolerance, providing insights for its practical use in viticulture.

## Introduction

Salt stress is a major global environmental factor that limits plant growth and crop productivity (Aragüés et al., 2015). When plants are subjected to salt stress, their osmotic regulation systems are severely disrupted, significantly reducing the efficiency of water and nutrient absorption from the soil (Liu et al., 2022). As the accumulation of inorganic salt ions in the plant, ion toxicity effects are triggered, leading to damage to the lipid membrane system, imbalance in ion homeostasis, and inhibition of photosynthesis. Ultimately, these effects result in leaf yellowing, stunted growth, and reduced yield. Therefore, enhancing plant stress resistance is therefore of critical importance (Liu et al., 2024).

Throughout evolution, plants have developed various regulatory mechanisms including morphological adaptations, osmotic substance regulation, antioxidant activity, ion transport, and mediation of endogenous hormone responses to cope with high salt stress and maintain normal growth and development (Zhao et al., 2020). In recent years, numerous studies have revealed that endogenous bioactive signaling molecules in plants play crucial roles in responding to environmental stresses, such as nitric oxide (Shams et al., 2019), ascorbic acid (Wang and Huang, 2019), and spermine (Ahanger et al., 2019). In addition, another group of substances -including neurotransmitters such as dopamine (Li et al., 2015), melatonin (Martínez et al., 2018), GABA (Zhu et al., 2019), serotonin (Lu et al., 2018), and acetylcholine (Braga et al., 2017) -also significantly contribute to plant adaptation to environmental stimuli.

Dopamine, a natural product of the catecholamine pathway, is a well-known neurotransmitter in mammals (Benes, 2001). In animals, it regulates neural activity in animals, promotes hormone secretion, enhances adaptive capacity, and facilitates metabolism and hormonal regulation. The discovery of dopamine in plants occurred later than in animals, and it has now been identified in the tissues and organs of over 40 plant species (Kimura, 1968). Dopamine exhibits potent antioxidant activity, with efficacy equivalent to ascorbic acid, gallocatechin, and epigallocatechin gallate (Liu et al., 2020). Under stress conditions, dopamine acts as a redox cofactor to regulate intracellular reactive oxygen species (ROS) scavenging systems and reduce ROS levels (Allen, 2003; Kanazawa and Sakakibara, 2000). In cucumbers, exogenous dopamine alleviates nitrate stress by reducing nitrate-N and ammonium-N accumulation through enhanced enzyme activity and upregulation of nitrogen metabolism-related gene expression (Lan et al., 2022). In watermelon, exogenous dopamine mitigates chilling stress damage by modulating osmotic regulation, antioxidant enzymes, and polyamine metabolism (Jiao et al., 2021). In rice, exogenous dopamine application enhances salt stress tolerance by regulating the expression of aquaporin protein *OsPIP*1-3 (Abdelkader et al., 2012). Additionally, dopamine promotes arbuscular mycorrhizal symbiosis through increased carbohydrate content. The synergistic interaction between arbuscular mycorrhizal fungi and dopamine helps maintain plant membrane stability and improves photosynthetic efficiency under high salinity, thereby enhancing plant salt tolerance (Gao et al., 2020a).

The grapevine is semi-sensitive to salinity; it can grow normally in soils with a salt content of 0.14 – 0.3%, exhibiting a relatively strong tolerance to salt-alkali stress. However, when the salt content exceeds a certain threshold (0.3%), grape growth and development are inhibited (Troncoso et al., 1999). Previous studies have shown significant differences in salt tolerance among different species of the genus *Vitis*. *Vitis* Champagne and Dog Ridge possess good salt resistance (Saritha et al., 2016); American species and *V. amurensis* × *V. vinifera* hybrids show moderate salt resistance (Mustapha et al., 2025); while *V. vinifera* and *V. vinifera* × American hybrids exhibit marked differences in salt resistance. A comprehensive evaluation of 20 table grape cultivars indicated that ‘Munage’, ‘Manaizi’, ‘Thompson Seedless’, and ‘Hotan Red’ are highly salt-tolerant cultivars, whereas ‘Kyoho’, ‘Red Muscat’, ‘Summer Black’, ‘Shine Muscat’, and ‘Sweet Sapphire’ are highly sensitive to salt (Zhang et al., 2026). Under salt-alkali stress, excessive accumulation of Na^+^ and Cl^-^ ions in the plant induces osmotic stress, ion toxicity, and oxidative damage, leading to inhibited photosynthesis, reduced growth, and decreased fruit quality, thereby seriously affecting grape yield and quality (Han and Li, 2024).

China is one of the countries with the largest saline-alkali land areas globally, currently possessing 99.13 million hectares of saline-alkali land which accounts for 10% of the country’s total land area (FAO, 2025). Soil salinization has become a major limiting factor constraining the sustainable development of the grape industry (Lu et al., 2022). This study aims to elucidate the physiological mechanisms by which dopamine enhances plant salt tolerance through investigating growth performance, ion homeostasis, ROS accumulation, and chlorophyll biosynthesis pathway. Concurrently, transcriptomic analysis has been conducted to explore the molecular mechanisms underlying dopamine-induced salt resistance in grapevines. These findings are expected to provide a mechanistic understanding of dopamine’s role in mitigating salt stress, thereby guiding its precise application in grape cultivation.

## Materials and methods

### Plant materials and treatments

In the experiment, one-year-old *Vitis vinifera* ‘Summer Black’ grape seedlings were grown in greenhouse under a 16 h light/8 h dark at 25 °C with LED lights (27 µmol m^-^² s^-^¹) to ensure full reproducibility. The plants were cultured in pots (18 cm diameter × 20 cm height) filled with sand. Uniformly growing seedlings with 7 – 8 leaves were selected. The experiment included a CK irrigated with 1/2 Hoagland nutrient solution, a salt treatment group irrigated with 200 mM NaCl solution, and dopamine treatment groups supplemented with salt solutions containing 0.2, 0.4, 0.6, 0.8, and 1.0 mM DA (NaCl + DA), respectively. Ten pots of grape plants with uniform growth were used per treatment (n=10). The treatment was initiated with a 50 mM NaCl solution, and the concentration was increased by 50 mM every 12 h until 200 mM NaCl was reached. The seedlings were watered with 2 L solution per pot every 3 days. Photosynthesis and chlorophyll fluorescence were measured on the 5th, 7th, and 10th days of treatment using photosynthesis instrument (CIRAS-3, PP systems, USA) and chlorophyll fluorometer (FMS2, Hansatech, UK), respectively. For these measurements, ten leaves with the 5th to 6th fully expanded from the top of the grape seedling were used (n=10). Morphological observation and measurement (plant height, stem diameter, fresh weight, and dry weight) were conducted on the 12th day of treatment for all seedlings (n=10). For other physiological indicators, the 2th to 3th fully expanded leaves from the top of the grape seedling were selected on day 12. Five seedlings with uniform growth were used per treatment (n=5).

### Determination of chlorophyll content and relative electrical conductivity in plants

Grape leaves were washed thoroughly with distilled water, and the main veins were removed before the leaves were cut into pieces. For chlorophyll measurement, 0.2 g of the prepared leaf sample was weighed and placed in 10 mL of 95% ethanol solution, then extracted in the dark for 48 h at room temperature. The absorbance of the extract was measured at wavelengths of 665 nm, 649 nm, and 470 nm using a spectrophotometer (UV2600, Shimadzu, Kyoto, Japan). The chlorophyll and carotene contents are calculated according to the method of Lichtenthaler and Wellburn (1983). For measurement of REC, the prepared leaf samples were placed in 10 mL of deionized water for 4 h to measure the initial electrical conductivity (R1). The samples were then boiled in a water bath for 20 minutes to determine the final electrical conductivity (R2). REC (%) = R1/R2×100%. Chlorophyll a (mg g^-1^) = (13.36D_665_ - 5.19D_645_)×V×W^-1^×10^-3^, Chlorophyll b (mg g^-1^) = (27.43D_645_ - 8.12D_663_)×V×W^-1^×10^-3^, Carotene (mg g^-1^) = (1000A_470_ - 2.13Ca - 97.64Cb)/209×V×W^-1^×10^-3^.

### Determination of Na^+^ and K^+^ content

Na^+^ and K^+^ contents were determined according to the method described by Munns et al. (2010). Grape leaves were placed in a 105 °C oven for 20 min for deactivation, followed by drying at 65 °C until a constant weight was achieved. The dried samples were ground, and 0.1 g of the powdered sample was mixed with 5 mL of sulfuric acid for 12 h. The next day, the mixture was heated on a digestion furnace until the solution turned a uniform dark brown. After cooling, 10 drops of 30% hydrogen peroxide were added, and the digestion continued for about 10 min. This digestion process was repeated 3 – 4 times until the solution became completely colorless and transparent. Finally, the solution was diluted to 100 mL with deionized water. The Na^+^ and K^+^ contents were measured using flame photometer(M410, Sherwood, UK)with the chromatographic grade NaCl or KCl for standard curves preparation.

### Determination of malondialdehyde, reactive oxygen species, and antioxidant enzymes activity

The grape leaves were selected for determination of membrane lipid peroxides, reactive oxygen species, and antioxidant enzyme activity levels. For each measurement, 0.2 g of leaf tissue was mixed with 1mL of extraction buffer (50 mM phosphate buffer solution, pH 7.4) and homogenize on ice. The mixture was centrifuged at 8400 g and 4 °C for 10 min, then collect the supernatant for assay according to the kit instructions (Beijing Solarbio Science & Technology Co., Ltd, China). Absorbance measurements were performed using spectrophotometer (UV2600, Shimadzu, Kyoto, Japan). MDA content was evaluated by the method of the TBA using the BC0020 kit (Hodges et al., 1999). The absorbance was measured at wavelengths of 532 nm and 600 nm. H_2_O_2_ contents was evaluated by the method of the titanium sulfate using a BC3595 kit (Satterfield and Bonnell, 1995). The absorbance was measured at a wavelength of 415 nm. O_2_^−^content was evaluated by the method of the hydroxylamine hydrochloride using a BC1290 kit (Elstner and Heupel, 1976). The absorbance was measured at a wavelength of 530 nm. Superoxide dismutase (SOD) activity was evaluated by the method of the NBT using the BC5160 kit (Giannopolitis and Ries, 1977). The absorbance was measured at a wavelength of 560 nm. Peroxidase (POD) activity was evaluated by the method of the guaiacol using the BC0090 kit (Doerge et al., 1997). The absorbance at 240 nm was measured every 30 seconds. Catalase (CAT) activity was evaluated by the method of the UV absorbance using the BC0200 kit (Johansson and Borg, 1988). The absorbance at 470 nm was measured at 30-second intervals. Glutathione peroxidase (GPX) activity was evaluated by the method of the 5,5’-Dithio-bis-(2-nitrobenzoic acid) (DTNB) using the BC1195 kit (Flohé and Günzler, 1984). The absorbance at 412 nm was recorded every 30 seconds. Glutathione reductase (GR) activity was evaluated by the method of the NADPH oxidation using the BC1160 kit (Foyer and Halliwell, 1976). The absorbance at 340 nm was measured at 60-second intervals.

### Transcriptome sequencing analysis

Leaves from the CK, salt, and salt + 0.4 mM DA-treated plants were sequenced using an Illumina high-throughput sequencing platform (Novogene Biotechnology Co., Ltd). Three biological replicates were set up, with three pots of grape plants showing uniform growth trends used per treatment (n=3). For data analysis, the trimmomatic software was used to remove sequencing adapters and filter out low-quality reads (Bolger et al., 2014) (https://phytozome-next.jgi.doe.gov/info/Vvinifera_v2_1). Clean reads were aligned to the *Vitis vinifera* v2.1 reference genome using the HISAT2 alignment software (Kim et al., 2015). Gene expression levels were normalized using the FPKM (Fragments per kilobase of transcripts per million mapped reads) method with the HTSeq-count software. Differentially expressed genes (DEGs) were screened using DESeq2 software with the criteria: q-value < 0.05 and |log2Foldchange| > 1. For Kyoto Encyclopedia of Genes and Genomes (KEGG) pathway analysis, pathway enrichment analysis for KEGG terms was performed on the list of DEGs using the enrichKEGG function from the cluster Profiler R package (v4.x.x) (Subramanian et al., 2005; Yu et al., 2012). The analysis used the most recent annotations for [*Vitis vinifera*] retrieved via the KEGG REST API. Significance of enrichment was determined using a hypergeometric test followed by Benjamini-Hochberg adjustment for multiple comparisons. Only pathways with an adjusted p-value < 0.05 were considered significantly enriched. Results are presented in a dop plot, where the size of the dot represents the number of DEGs in pathway, and the x-axis represents the Rich Factor. For visualization of gene across samples, a heatmap was generated using the pheatmap package. The input was variance-stabilized transformed count data of the top most variable significant DEGs. Expression values were transformed to Z-scores to show relative expression per gene.

### RNA analysis

Total RNA was extracted from grape leaves using the E.Z.N.A. Total RNA Kit I (OMEGA Bio-tek, Inc., USA). The quality and concentration of the total RNA samples were assessed by 2% (w/v) agarose gel electrophoresis and a NanDrop ND-1000 spectrophotometer (Thermo Scientific, Waltham, MA, USA), with A260/A280 and A260/A230 ratios exceeding 1.8. The first-strand cDNA was synthesized using SMART MMLV Reverse Transcriptase (TaKaRa, Beijing, China). All RT-qPCR experiments were performed using the Applied Biosystems PCR instrument (ABI, Los Angeles, CA, USA). with the flowing cycling conditions: 1 cycle of 95 °C for 30 s, followed by 40 cycles of 95 °C for 10 s, and 60 °C for 30 s. The reaction system (20 μL) was as follows: 10 μL 2×SYBR Green I Master Mix, 0.8 μL (each) specific primers, 2 μL (100 ng) cDNA, and 6.4 μL of sterile water. The relative mRNA ratios were calculated using the 2 ^−δδCT^ method (Livak and Schmittgen, 2001). Each sample was tested in three technical replicates, and the average value was used for RT-qPCR analysis. The primers used for qPCR are listed in **Supplementary Table 1**.

### Data analysis

All data were subjected to one-way analysis of variance (ANOVA) with Duncan’s multiple range test at *p* < 0.05 using SPSS 27.0 (SPSS Inc., Chicago, IL, USA). All figures were expressed as mean ± standard deviation (SD) based on triplicate repetitions.

## Results

### Exogenous dopamine promotes the growth of grape aerial parts under salt stress

**Figure 1:**
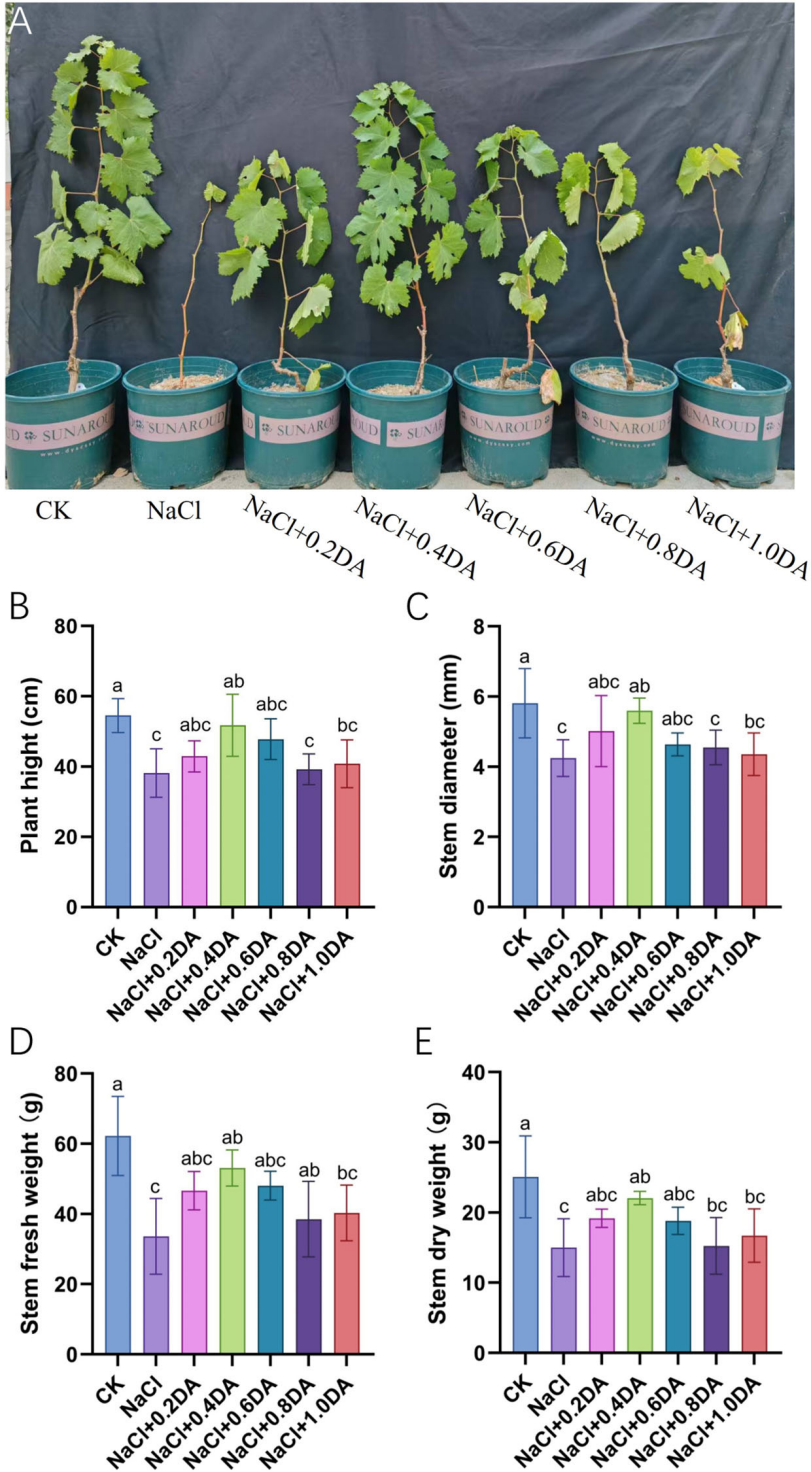
Amelioration effect of dopamine on the growth in grape seedlings under salt stress. **(A)** Photographic image of grape seedlings under NaCl or NaCl + DA treatments; DA: Dopamine **(B)** Plant height; **(C)** Stem diameter; **(D)** Fresh stem weight; **(E)** Dry stem weight. CK: Control group; NaCl: Salt treatment with 0.2 mol NaCl; NaCl+0.2 (0.4, 0.6. 0.8, 1.0) DA: Salt treatment combined with exogenous application of 0.2, 0.4, 0.6. 0.8, or 1.0 mM dopamine. Values represent mean ± SD (n=10). Different lowercase letters on the error bars indicate significant differences at *p* < 0.05 according to the Duncan’ test.

After 12 days of treatment with 200 mM NaCl, grape seedlings exhibited severe leaf yellowing and abscission (**Figure 1A**), with plant height and stem diameter reduced to 70.05% and 73.10% of CK levels, respectively (**Figures 1B, C**). Exogenous application of dopamine markedly alleviated salt-induced growth inhibition, with the 0.4 mM dopamine showing the most pronounced effects: plant height and stem diameter were maintained at 94.99% and 96.33% of the CK levels, respectively. Measurements of aboveground biomass revealed that salt treatment alone reduced plant fresh weight and dry weight to 54.00% and 59.77% of the CK levels, respectively. Exogenous dopamine at different concentrations promoted increases in fresh and dry weights of grape stems, with 0.4 mM dopamine again showing the most significant effects - fresh and dry weights reached 85.29% and 87.98% of CK levels, respectively. These results demonstrate that exogenous dopamine effectively alleviates salt stress-induced growth inhibition and promotes aboveground biomass accumulation in grapevines.

### Exogenous dopamine promotes the growth of grape roots under salt stress

**Figure 2:**
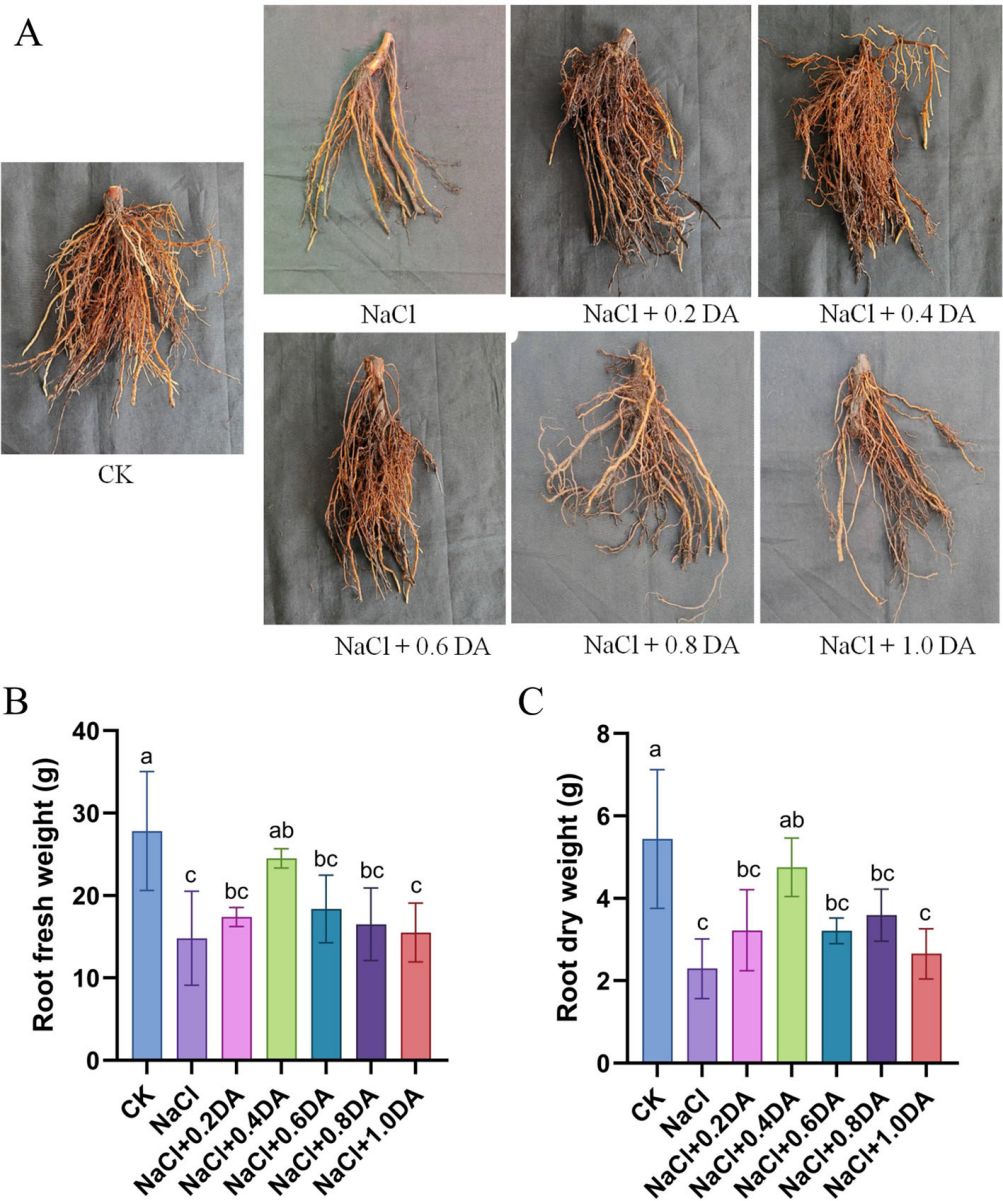
Root phenotypes of grapevines under salt stress with dopamine treatments. **(A)** Photographic image of grape roots under NaCl or NaCl + DA treatments, DA: Dopamine; **(B)** Root fresh weight; **(C)** Root dry weight. Values represent mean ± SD (n=10). Different lowercase letters on the error bars indicate significant differences at *p* < 0.05 according to the Duncan’ test.

Salt stress significantly inhibits the growth of grape roots. After 12 days salt treatment alone, root fresh weight and dry weight were only 53.28% and 42.15% of the CK levels, respectively (**Figure 2**). Exogenous application of dopamine at different concentrations promoted the increase of fresh and dry weights in grape seedling roots, with 0.4 mM dopamine treatment shows the most pronounced effect -reaching 88.07% and 87.37% of the CK levels, respectively. As shown in **Table 1**, treatments with various dopamine concentrations significantly improved root architecture parameters, with 0.4 mM dopamine again demonstrating the most remarkable effects. Compared to salt stress treatment alone, the 0.4 mM dopamine treatment increased total root length and root surface area by 73.63% and 93.29%, respectively; elevated average root diameter and root volume by 140% and 118.99%; and boosted root tip number and lateral root number by 70.87% and 74.26%, respectively. These results indicate that exogenous dopamine can effectively promote grape root development under salt stress.

**Table 1:** Effects of different concentrations of DA on root architecture parameters of grape under salt stress.

Treatment	Root length (cm)	Surface area (cm^2^)	Average diameter (mm)	Root volume (cm^3^)	Tips	Forks
CK	2414.1 ± 367.80 a	775.65 ± 99.56 a	3.06 ± 0.71 a	20.28 ± 3.99 a	15643 ± 2280 a	15123.33 ± 2237.79 a
NaCl	1271.5 ± 355.87 a	350.53 ± 47.16 b	1.15 ± 0.2 b	7.74 ± 1.08 a	8198.67 ± 378.88 a	7714.67 ± 1029.74 a
NaCl+0.2 Da	1868.2 ± 229.65 a	552.4 ± 73.71 ab	2.14 ± 0.3 ab	13.12 ± 2.59 a	12311.67 ± 598.17a	11831 ± 2093.16 a
NaCl+0.4 Da	2207.9 ± 379.63 a	677.55 ± 87.24 ab	2.76 ± 0.38 a	16.95 ± 2.75 a	14008 ± 1758.18 a	13443.67 ± 1529.41 a
NaCl+0.6 Da	1862.5 ± 333.52 a	597.38 ± 78.76 ab	2.07 ± 0.35 ab	15.74 ± 2.16 a	13925 ± 1554.9 a	11010.33 ± 1442.64 a
NaCl+0.8 Da	1839.1 ± 200.51 a	621.53 ± 78.56 ab	1.93 ± 0.36 ab	15.64 ± 2.77 a	10526.67 ± 1671.63 a	10338 ± 1537.16 a
NaCl+1.0 Da	1460.3 ± 118.09 a	422.15 ± 45.25 ab	1.87 ± 0.35 ab	10.39 ± 1.96 a	8462 ± 1114.34 a	9597 ± 1069.81 a

Different lower case letters on the error bars indicate significant differences at p<0.05 according to the Duncan’ test.

### Exogenous dopamine promotes grape photosynthesis under salt stress

**Figure 3:**
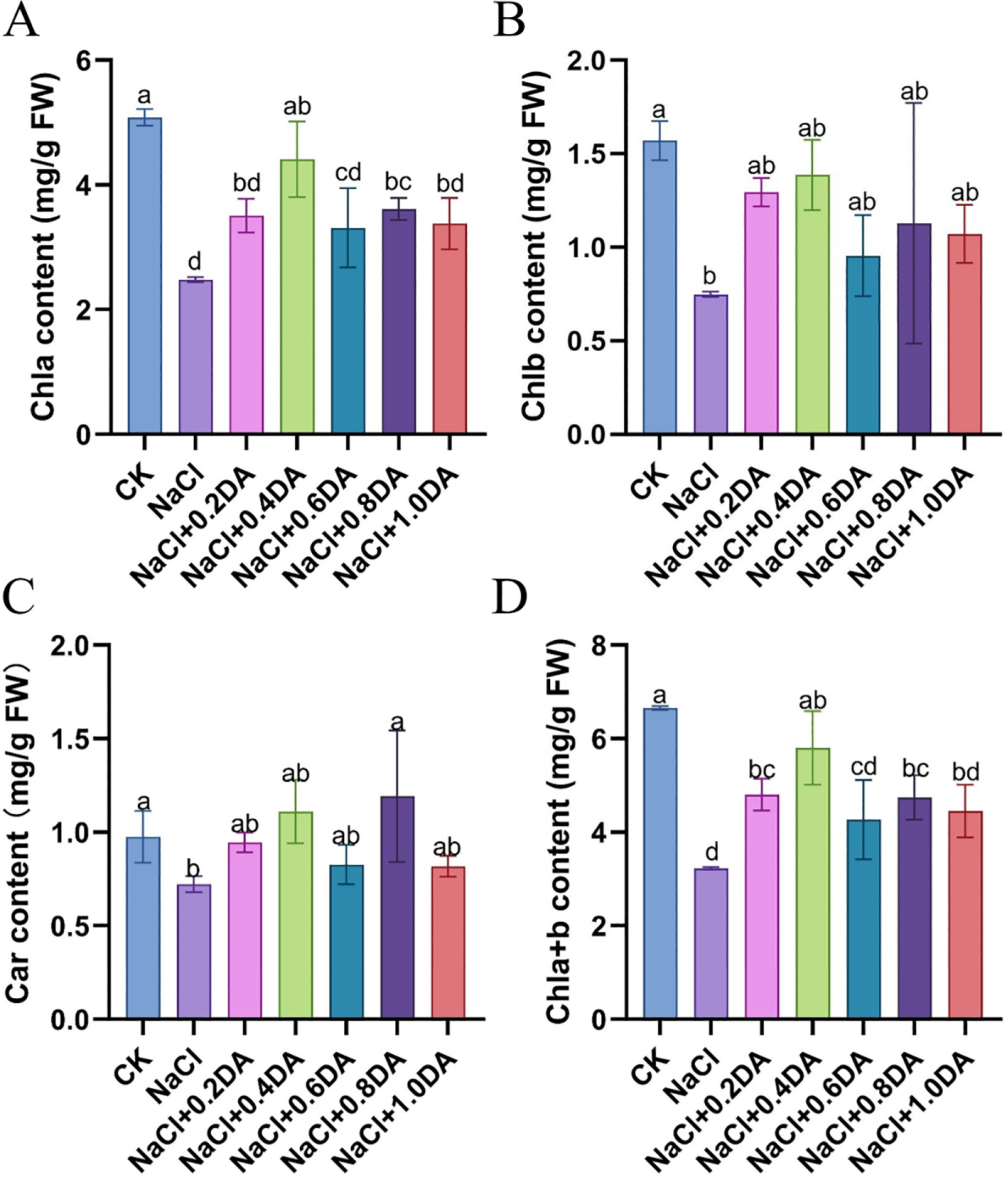
Effects of dopamine on chlorophyll content in grapevines under salt stress. **(A)** Chlorophyll a (Chl a) content; **(B)** Chlorophyll b (Chl b) content; **(C)** Carotenoid (Car) content; **(D)** Total chlorophyll content. Values represent mean ± SD (n=5). Different lowercase letters on the error bars indicate significant differences at *p* < 0.05 according to the Duncan’ test.

The content of chlorophyll directly affects the photosynthetic efficiency of plants. As shown in **Figure 3**, after 12 days of salt stress, the chlorophyll a, chlorophyll b, total chlorophyll, and carotenoid contents were only 48.79%, 47.74%, 48.55% and 63.81 of the CK levels, respectively. Compared with the salt treatment alone, the application of different concentrations of dopamine significantly increased the contents of chlorophyll. Among these, 0.4 mM dopamine showed the most remarkable effect, with chlorophyll a, chlorophyll b, total chlorophyll, and carotenoid contents reaching 86.80%, 88.35%, 87.17%, and 113.83% of the CK levels, respectively.

**Figure 4:**
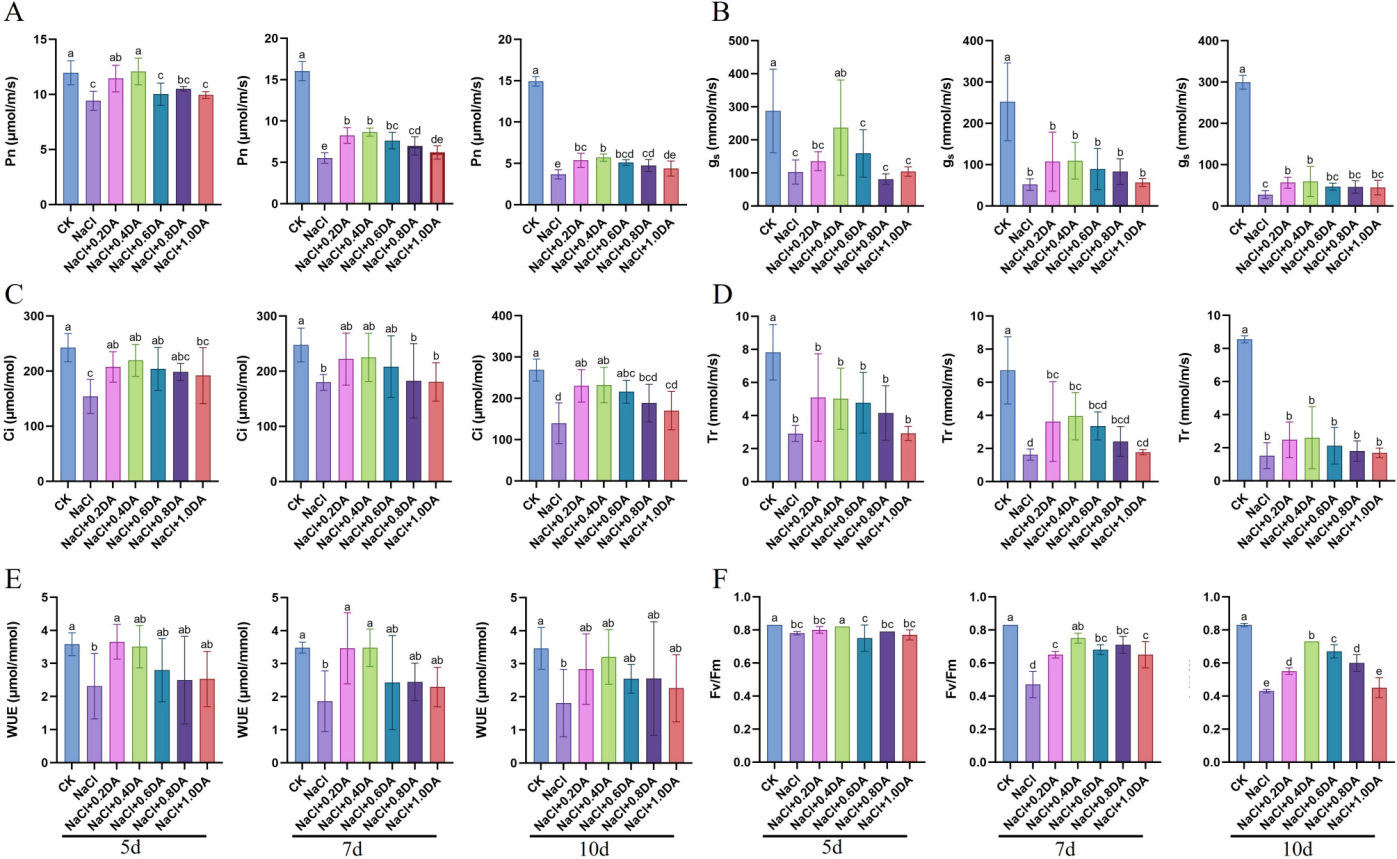
Effects of dopamine on photosynthetic parameters in grapevines under salt stress. Photosynthesis and chlorophyll fluorescence were measured on the 5th, 7th, and 10th days of treatment respectively. **(A)** Net photosynthesis rate (Pn); **(B)** Stomatal conductance (Gs); **(C)** Intercellular CO_2_ concentration (Ci); **(D)** Transpiration rate (Tr); **(E)** Water utilization rate (WUE); **(F)** Maximum photochemical efficiency of PSII (Fv/Fm). Values represent mean ± SD (n=10). Different lowercase letters on the error bars indicate significant differences at *p* < 0.05 according to the Duncan’ test.

Salt stress significantly inhibited the photosynthetic physiological indices of grape seedlings. As shown in **Figure 4**, after 5 days of salt treatment alone, the net photosynthetic rate (Pn), stomatal conductance (Gs), intercellular CO_2_ concentration (Ci), transpiration rate (Tr), and water use efficiency (WUE) were significantly lower than those of the CK and continued to decline with prolonged treatment duration. By day 10, these indices had decreased to 24.66%, 9.15%, 51.94%, 17.75% and 52.28% of the CK levels, respectively. Exogenous dopamine treatment significantly alleviated the decline in these indices, with the 0.4 mM concentration showing the most pronounced effect: at 10 days of salt treatment, its Pn, Gs, Ci, Tr, and WUE maintained at 38.20%, 19.71%, 86.44%, 30.41%, and 92.33% of the CK levels, respectively; Meanwhile, the variation trend of the maximum photochemical efficiency of PSII (Fv/Fm) was consistent with that of photosynthesis. After 10 days of salt treatment, the Fv/Fm value was only 51.81% of the CK levels, whereas the 0.4 mM dopamine treatment group maintained 87.95% of the CK levels. Collectively, these findings collectively demonstrate that dopamine at appropriate concentrations can alleviate the adverse effects of salt stress on photosynthetic performance in grapevines.

### Dopamine maintain the ion balance in grape under salt stress

**Figure 5:**
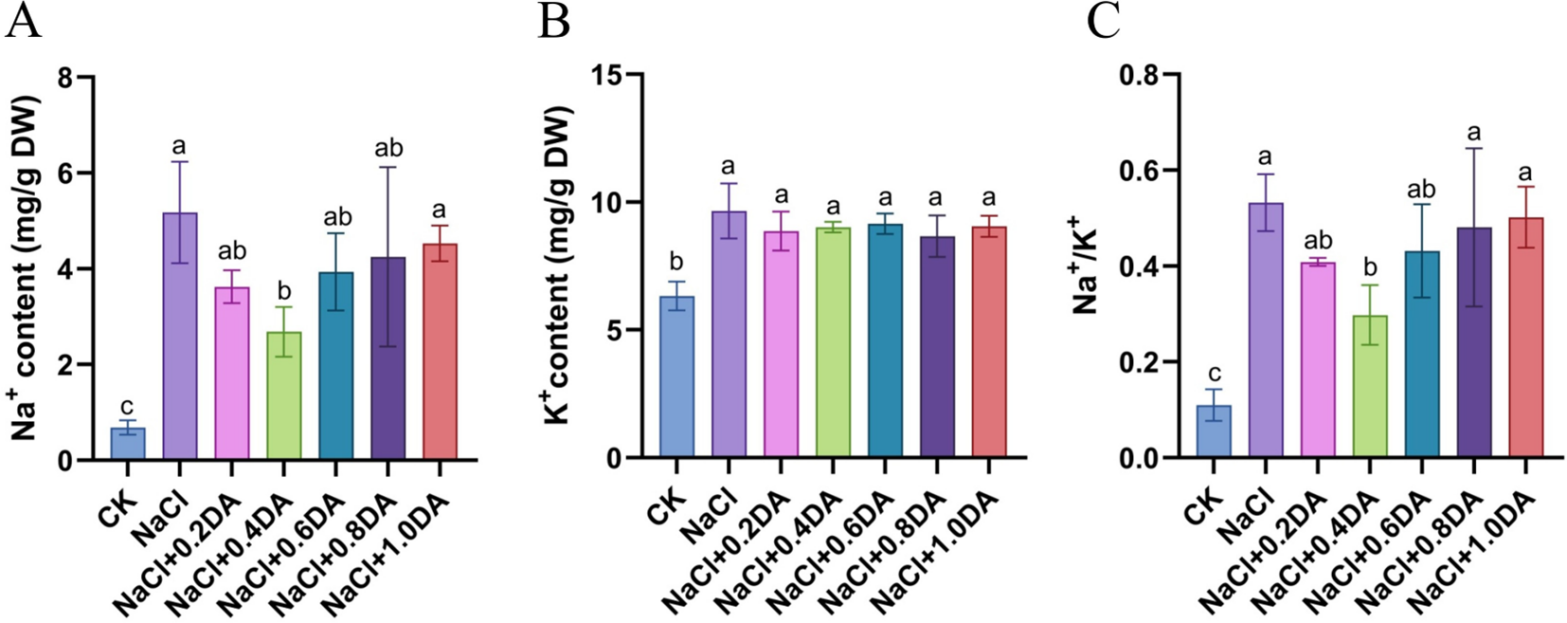
Effects of dopamine on ion content in grapevines under salt stress. **(A)** Na^+^ content; **(B)** K^+^ content; **(C)** Na^+^/K^+^ ratio. Values represent mean ± SD (n=5). Different lowercase letters on the error bars indicate significant differences at *p* < 0.05 according to the Duncan’ test.

Under salt stress, plant ion homeostasis can be characterized by changes in Na^+^ and K^+^ content. As shown in **Figure 5**, salt treatment significantly increased Na^+^ content, K^+^ content, and Na^+^/K^+^ ratio in grape seedling leaves, reaching 756.50%, 152.58%, and 483.91% of the CK levels, respectively. Exogenous dopamine treatment effectively regulated ionic balance. Compared to salt treatment alone, the 0.4 mM dopamine treatment reduced Na^+^ content by 48.17% maintained relatively stable K^+^ content, and significantly decreased the Na^+^/K^+^ ratio to 55.98% of that in the salt-treated group. These results demonstrate that exogenous dopamine application under salt stress can reduce Na^+^ accumulation and maintain a stable Na^+^/K^+^ ratio in grape plants.

### Dopamine alleviate ROS damage in grape under salt stress

**Figure 6:**
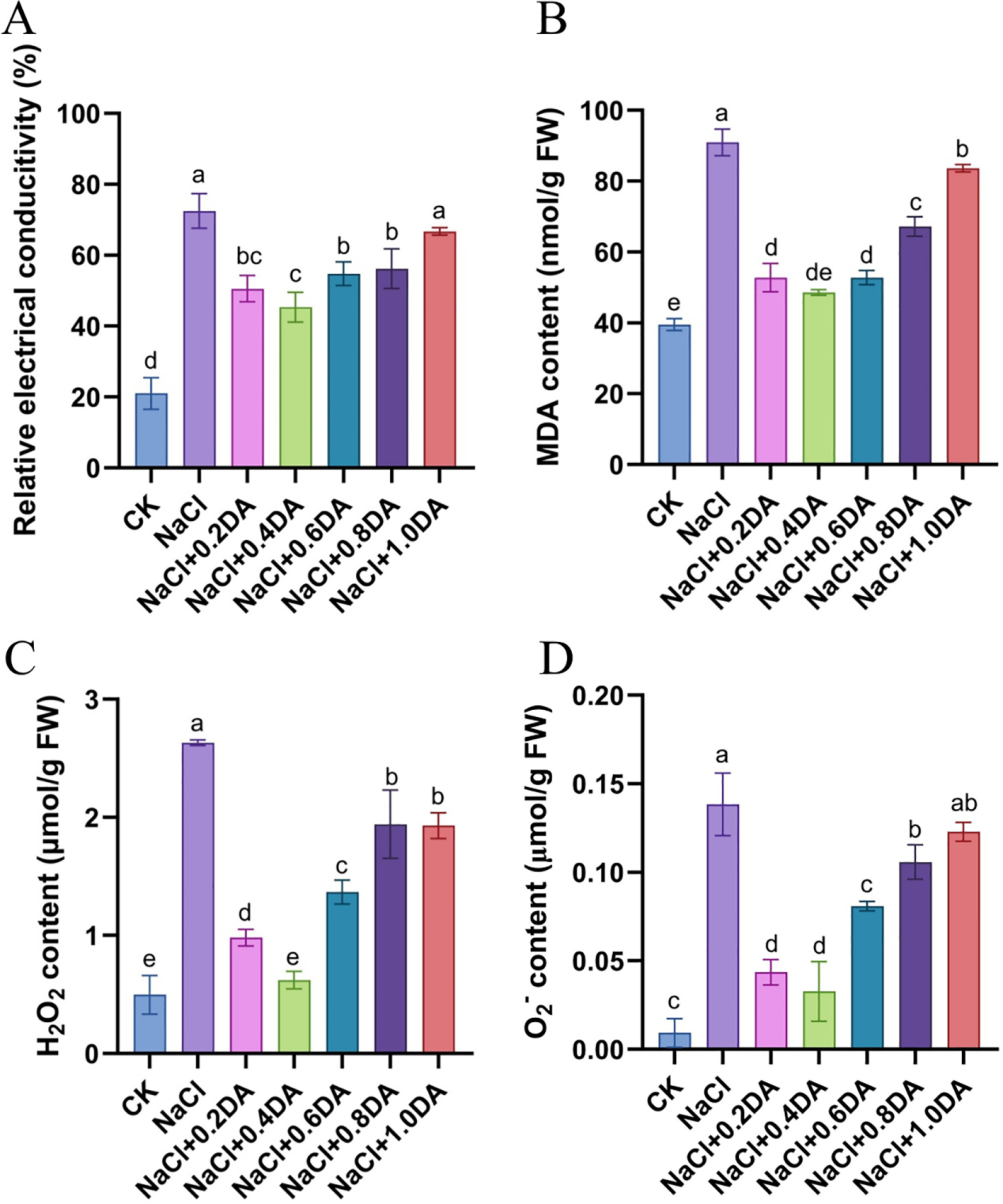
Effects of dopamine on MDA and ROS content in grapevines under salt stress. **(A)** Relative conductivity; **(B)** MDA content; **(C)** H_2_O_2_ content; **(D)** O_2_^−^ content. Values represent mean ± SD (n=5). Different lowercase letters on the error bars indicate significant differences at *p* < 0.05 according to the Duncan’ test.

Salt treatment significantly increased the REC of grape seedlings. The REC in the salt-alone treatment was 345.2% that of the CK level. Application of exogenous dopamine alleviated ion leakage; in the 0.4 mM dopamine treatment, in the REC was 69.8% that of in the salt treatment (**Figure 6A**). Meanwhile, the contents of MDA and ROS were measured. As shown in **Figures 6B–D**, salt treatment alone significantly elevated the MDA, H_2_O_2_, and O_2_^−^ levels in grape leaves, reaching approximately 228.9%, 528.5%, and 1489.2% of the CK levels, respectively. Treatments with different concentrations of exogenous dopamine decreased ROS accumulation. Under 0.4 mM dopamine treatment, the MDA, H_2_O_2_, and O_2_^−^ contents were only 53.43%, 23.63% and 23.61% of the salt treatment alone, respectively. These results demonstrate that dopamine application alleviates electrolyte leakage and cell membrane damage in grape seedlings under salt stress.

### Dopamine activate the antioxidant enzyme activities in grape under salt stress

**Figure 7:**
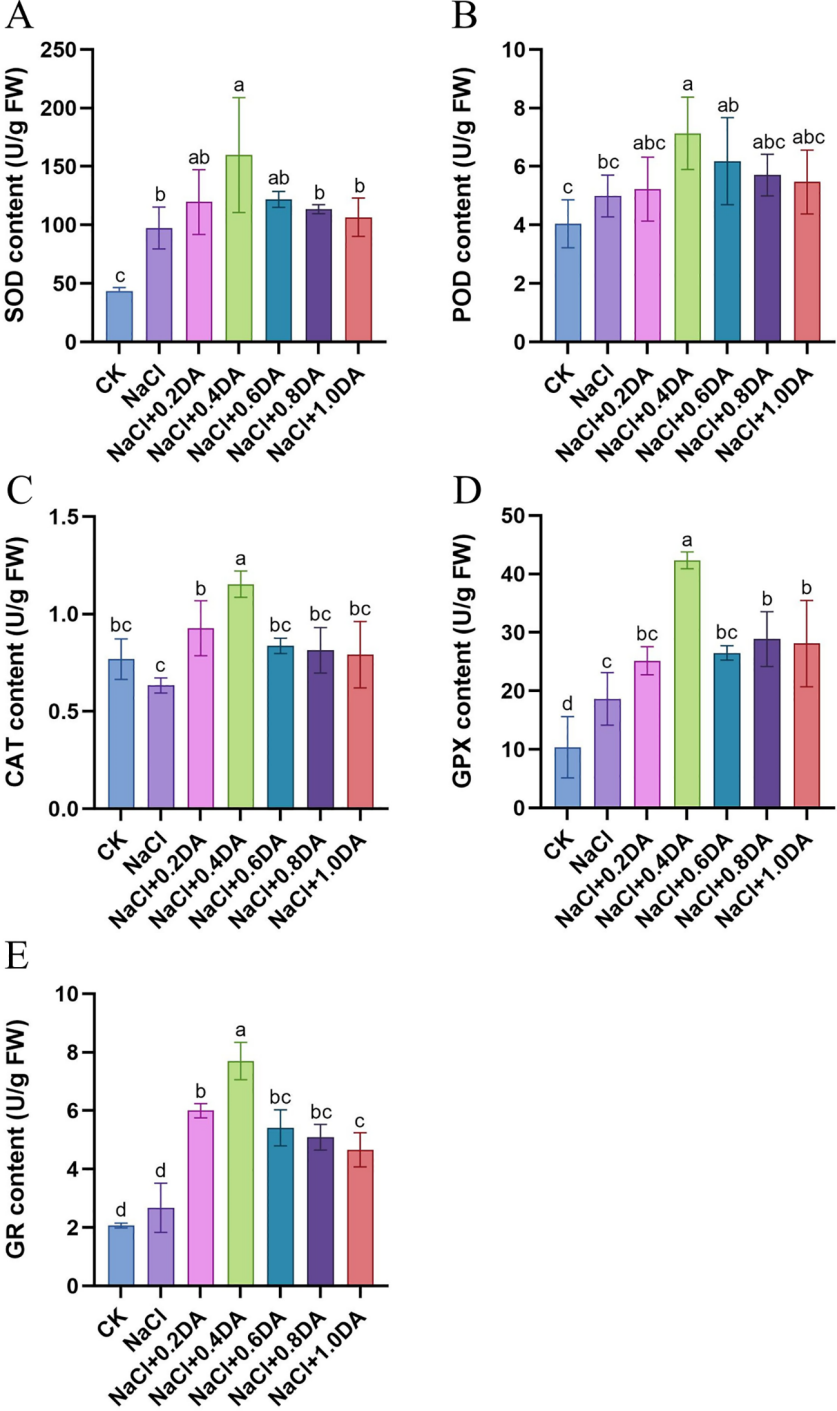
Effects of dopamine on antioxidant activities of grapevines under salt stress. **(A)** SOD activity; **(B)** POD activity; **(C)** CAT activity; **(D)** GPX activity; **(E)** GR activity. Values represent mean ± SD (n=5). Different lowercase letters on the error bars indicate significant differences at *p* < 0.05 according to the Duncan’ test.

Under stress conditions, plants alleviate oxidative damage caused by adversity stress by scavenging excess reactive oxygen species through their antioxidant enzyme system. As shown in **Figure 7**, salt stress increased the activities of SOD, POD, GPX and GR in grape leaves by 122.69%, 23.59%, 79.49%, and 29.47% respectively compared to the CK levels, while, the CAT activity decreased to 82.4% of the CK levels. After dopamine treatment, the activities of SOD, POD, CAT, GPX, and GR were significantly higher than those under salt stress alone, exhibiting an initial increase followed by a downward trend with increasing dopamine concentrations. Specifically, at a dopamine concentration of 0.4 mM DA, the activities of SOD, POD, CAT, GPX and GR reached their peak levels, showing increases of 64.05%, 42.86%, 82.14%, 127.14% and 187.31%, respectively, compared to the salt-stressed group. These results indicate that appropriate dopamine concentrations can effectively mitigate salt-induced oxidative damage by activating the antioxidant enzyme system.

### Screening of DEGs in grape under salt stress based on transcriptome analysis

A transcriptome analysis was conducted on grape leaves treated with salt alone and 0.4 mM dopamine (NaCl + 0.4 DA) for 3 days. After removing adapter sequences, unknown splice sequences, and low-quality sequences, a total of 63.88G of clean base data (150 bp reads per sample) was obtained, with an average of 7.10G per sample (**Supplementary Table 2**). The sequencing quality metrics showed Q20% ≥98% and Q30% ≥95%, and GC content ranging between 44.59% and 46.05%, providing a robust data foundation for subsequent analyzes.

Differential gene expression analysis was performed based on the screening thresholds of padj<0.05 and |log2Fold Change| >1 (**Supplementary Figure 1**). Volcano plot analysis revealed 4951 DEGs between the CK and salt alone treatment group (NaCl), with 2318 up-regulated and 2623 down-regulated genes; 4937 DEGs between the CK and salt + dopamine group (DA), with 2297 up-regulated and 2640 down-regulated genes; and 420 DEGs between the salt treatment group (NaCl) and salt + dopamine group (DA), including 168 up-regulated and 252 down-regulated genes.

### KEGG analysis of DEGs

**Figure 8:**
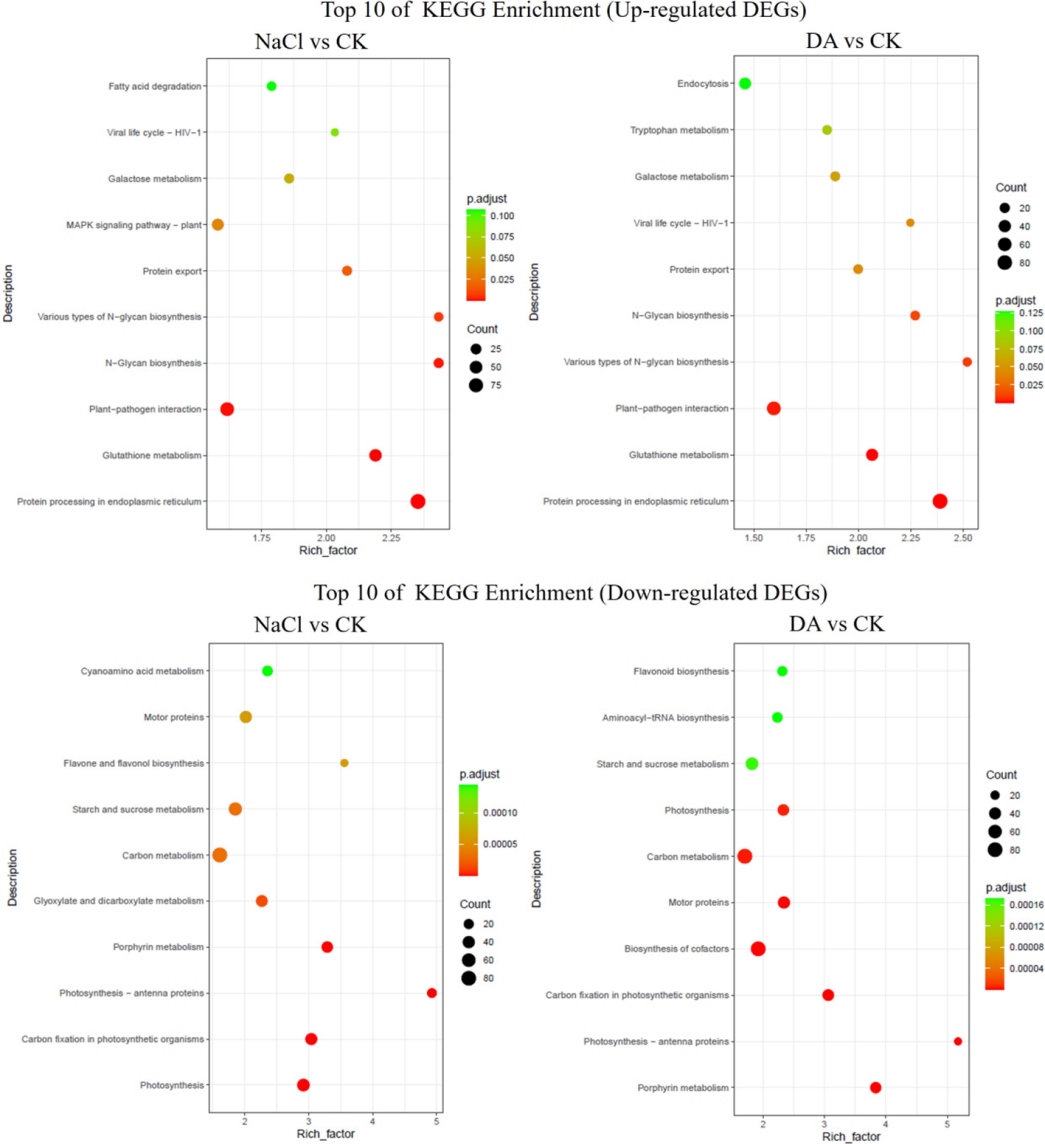
The KEGG enrichment analysis of differentially expressed genes (DEGs) in NaCl vs CK and DA vs CK. The top 10 KEGG enrichment pathways are shown for up-regulated or down-regulated DEGs in NaCl vs CK and DA vs CK.

**Figure 9:**
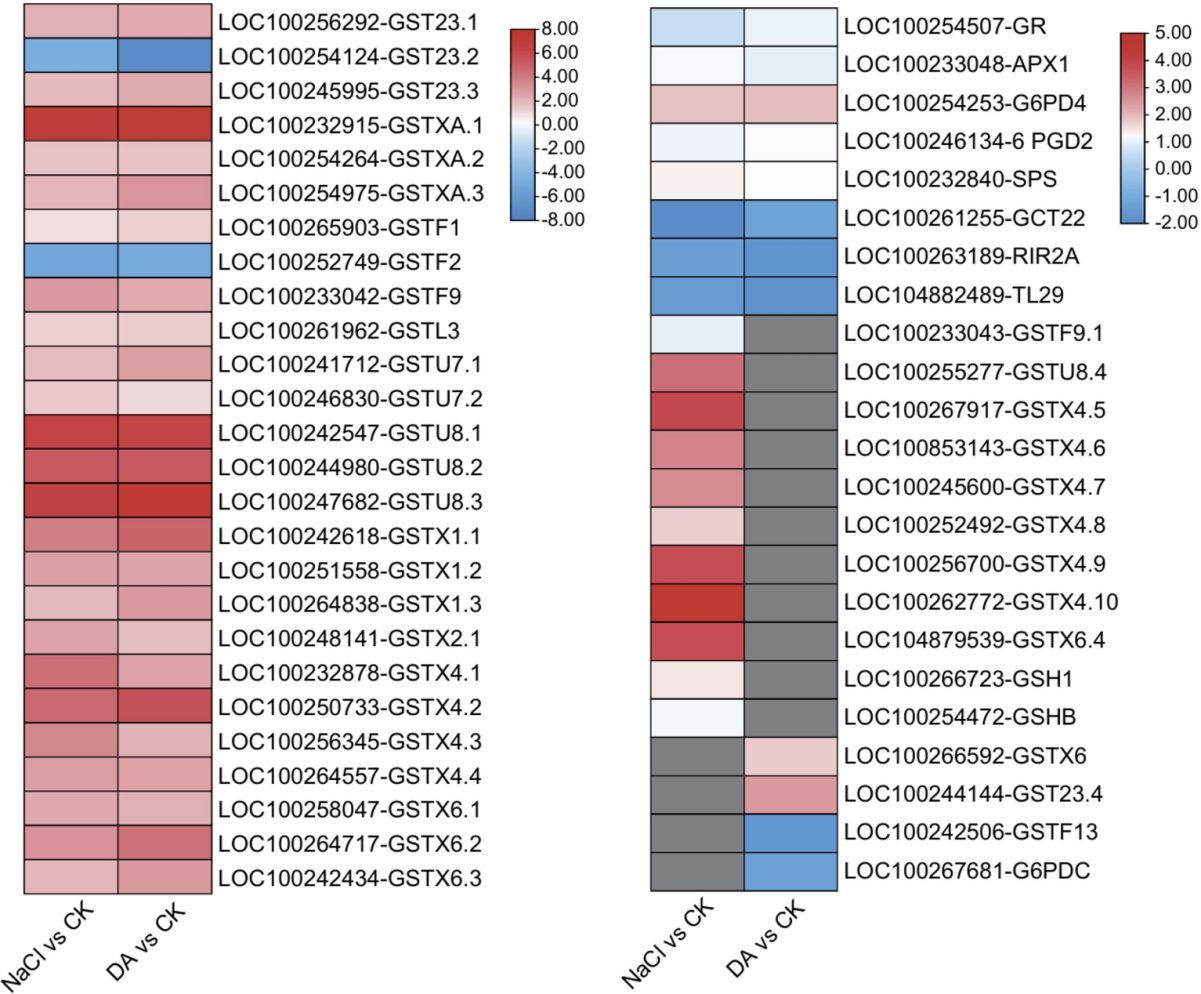
Expression analysis of differentially expressed genes (DEGs) involved in the “glutathione metabolism” pathway. Gray indicates non-significant expression of unigenes.

In the KEGG analysis of DEGs, the up-regulated genes in the NaCl vs. CK and DA vs. CK showed approximately 90% overlap in the top 10 enriched metabolic pathways. These pathways were primarily enriched in protein processing in the endoplasmic reticulum, glutathione metabolism, plant-pathogen interaction, N-Glycan biosynthesis, various types of N-Glycan biosynthesis, protein export, and galactose metabolism (**Figure 8**). Previous studies have shown that glutathione metabolism plays a critical role in plant stress response. This pathway includes 43 DEGs from NaCl vs CK and 38 DEGs from DA vs CK, such as glutathione S-transferases, glutathione peroxidases, glutathione reductases, ascorbate peroxidases, glutamate-cysteine ligases, spermine synthases, proline enzymes, and gluconate dehydrogenases (**Figure 9**). Among these, 32 GST-related DEGs from NaCl vs CK and 26 GST-related DEGs from DA vs CK were significantly up-regulated, which account for 94% and 90% of the total GST-related DEGs in each group, respectively. Notable up-regulated genes included LOC100232915 (*GSTXA*) with log2(Fold Change) values of 7.122 and 7.009, LOC100247682 (*GSTU8*) with log2(Fold Change) values of 7.411 and 6.605, LOC100242547 (*GSTU8*) with log2(Fold Change) values of 6.361 and 6.466 (**Figure 9**).

**Figure 10:**
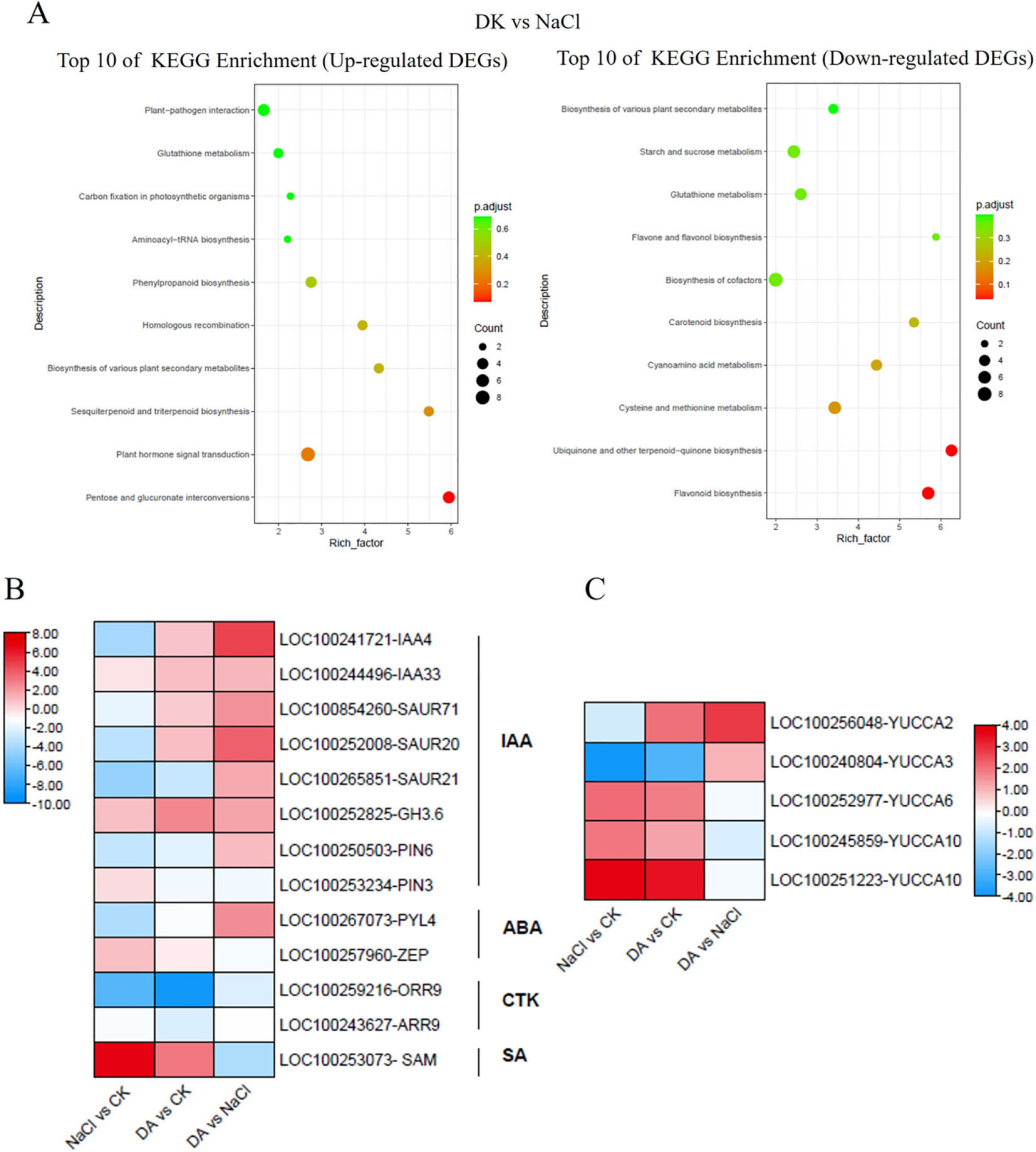
The KEGG enrichment analysis of differentially expressed genes (DEGs) in DA vs NaCl group. Top 20 KEGG enrichment pathways for up-regulated DEGs in Da vs NaCl group. **(A, B)** Expression analysis of the DEGs involved in the “plant hormone signal transduction” pathway. **(C)** Expression analysis of the DEGs related to the IAA synthase gene *YUCCA*.

In the KEGG analysis of DEGs between the Da vs NaCl groups, the up-regulated genes were primarily enriched in pathways including pentose and glucuronate interconversions, plant hormone signal transduction, sesquiterpenoid biosynthesis, biosynthesis of various plant secondary metabolites, homologous recombination, phenylpropanoid biosynthesis, plant-pathogen interaction, and glutathione metabolism (**Figure 10A**). The plant hormone signal transduction pathway contained 13 DEGs, comprising 8 up-regulated and 5 down-regulated genes (**Figure 10B**). Among the up-regulated genes, 7 DEGs were associated with IAA signal transduction (auxin-responsive protein *IAA4*, *IAA33*, auxin-responsive protein *SAUR21*, *SAUR71*, auxin efflux carrier component *PIN6*, indole-3-acetic acid-amido synthetase *GH3.6*), and 1 was ABA signal transduction-related gene *PYL4*. Additionally, we identified 5 DEGs related to IAA synthase genes in the NaCl vs CK: three up-regulated DEGs (*YUCCA6*, *10*, *10*) and two down-regulated DEGs (*YUCCA2*, *3*). In the DA vs. CK, four DEGs were up-regulated (*YUCCA2*, *6*, *10*, *10*) while one DEG was down-regulated (*YUCCA3*). In the DA vs NaCl group, two DEGs were up-regulated (*YUCCA2*, *3*) (**Figure 10C**). KEGG analysis of down-regulated genes revealed primary enrichment in flavonoid biosynthesis, ubiquinone and other terpenoid-quinone biosynthesis, cysteine and methionine metabolism, cyanoamino acid metabolism, and carotenoid biosynthesis.

### Validation of transcriptome data by RT-qPCR

**Figure 11:**
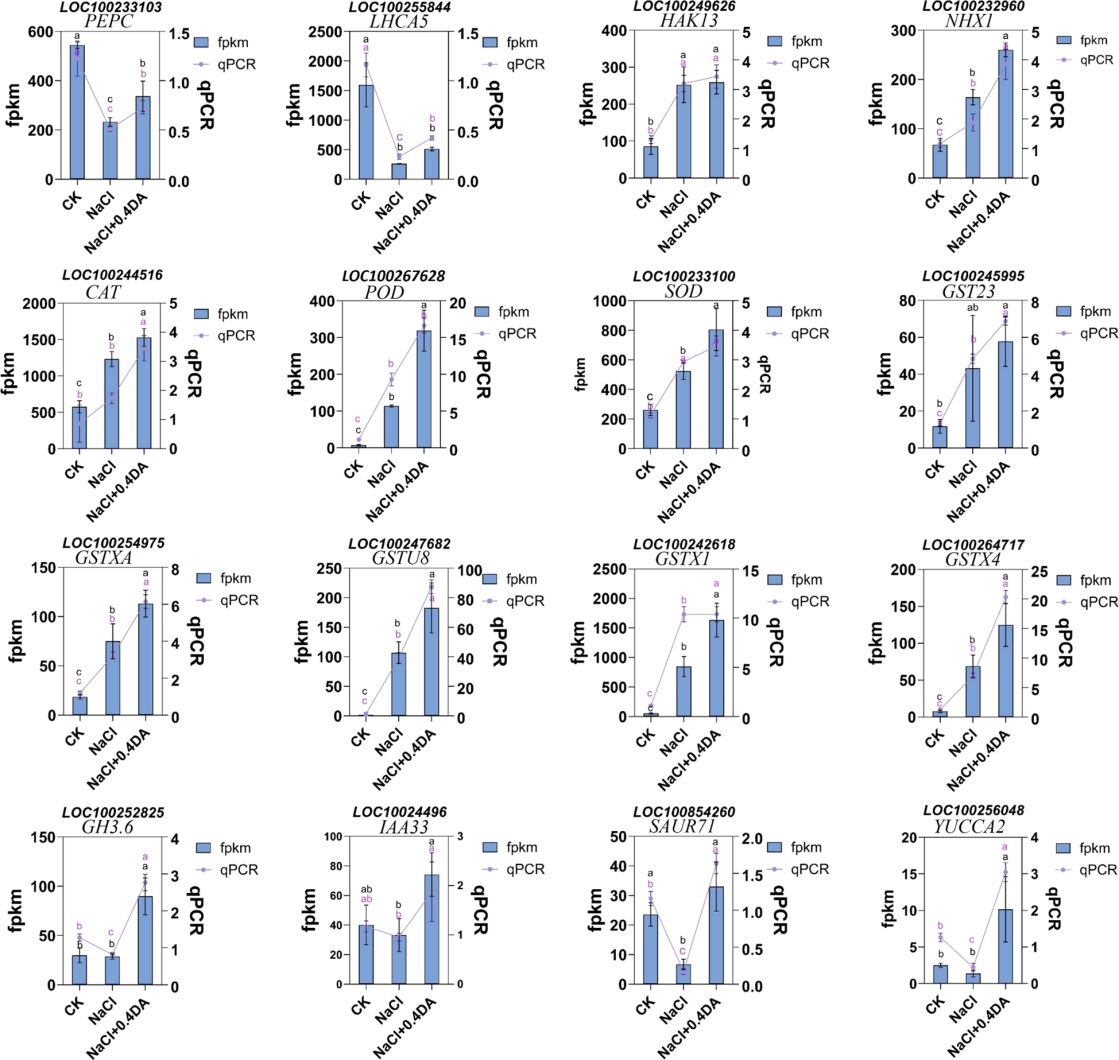
RT-PCR validation of RNA-seq data. Expression profiles of selected genes as determined by RNA-seq and qRT-PCR.

To verify the reliability of the transcriptome data, the RT-qPCR was performed to validate the expression patterns of 16 randomly selected candidate genes, including photosynthesis-related genes (*PEPC*, *LHCA5*), ion transport-related genes (*HAK13*, *NHX1*), antioxidant-related genes (*SOD*, *POD*, *CAT*, *GSTXA*, *GSTU8*, *GSTX1*, *GSTX4*), and auxin signaling pathway-related genes (*GH3.6*, *IAA33*, *SAUR71*, *YUCCA2*). The results showed that both RT-qPCR and transcriptome sequencing data exhibited highly consistent expression trends (**Figure 11**), indicating that the transcriptome sequencing results are reliable.

## Discussion

As an important fruit, the grapevine is cultivated worldwide. However, soil salinization constrained the sustainable development of the grape industry (Lu et al., 2022). According to the latest 2025 report from the Food and Agriculture Organization of the United Nations (FAO), the global area of saline-alkali land is approximately 1.381 billion hectares, accounting for 10.7% of the world’s arable land. Australia, Argentina, Kazakhstan, and China are among the most severely affected countries (FAO, 2025). Therefore, improving the salt tolerance of grapevines is crucial for the grape industry.

Dopamine, a catecholamine compound, not only serves as a precursor for the synthesis of numerous alkaloids but also acts as an important endogenous signaling molecule involved in plant responses to internal and external environmental signals, particularly stress conditions such as cold (Jiao et al., 2021), drought (Gao et al., 2020b), salt (Abdelkader et al., 2012), heavy metal (Cao et al., 2023) and so on. However, its role in alleviating stress in grapes has not yet been explored. In this study, exogenous different concentrations of dopamine application significantly alleviated leaf yellowing and wilting under high-salt stress, increased stem and root dry/fresh weights, and enhanced overall plant biomass compared with salt stress alone. These results indicate that exogenous dopamine can effectively enhance salt stress tolerance in grapes. Notably, we found that the effect of dopamine on improving plant salt tolerance is dose-dependent, with the application of 0.4 mmol dopamine being the most effective. This phenomenon has also been observed in apples, where compared to other concentrations, exogenous application of 0.1 mmol dopamine most significantly improved alkaline tolerance (Jiao et al., 2019). Traditional toxicology holds that “the dose makes the poison”. Low doses of γ-rays can enhance salt stress resistance in rice, while high doses can damage physiological indicators such as antioxidant enzyme (Macovei et al., 2014). Moreover, in apple and watermelon, although dopamine can enhance plant tolerance to drought and cold stresses, different concentrations of dopamine have been shown to cause significant differences in photosynthesis, ion transport and antioxidant enzymes (Gao et al., 2020b; Jiao et al., 2021). Based on these findings, we hypothesized that the dose-dependent salt tolerance improvement observed in our study may be mediated by differential regulation of these physiological indicators.

Photosynthesis is a key physiological and metabolic process in plant growth and development, changes in its activity serve as an important physiological indicator for assessing plant stress resistance (Chauhan et al., 2023). Salt stress significantly inhibits light energy capture, conversion, and electron transport efficiency in various crops such as cotton (Zhang et al., 2014), muskmelon (Jin et al., 2019), and tomato (Dou et al., 2025), leading to chloroplast structural damage and functional disruption. In this study, salt stress markedly reduced chlorophyll content and photosynthetic parameters in grape leaves, while exogenous dopamine treatment effectively alleviated this inhibition, with the 0.4 mM DA treatment group showing the greatest alleviation. Similar results have been observed in apple, where dopamine enhanced the photosynthetic activity (Jiao et al., 2019). In grapevine, melatonin increased the Ci, Fv/Fm, and chlorophyll content (Li et al., 2024). Light capture and carbon fixation play a crucial role in photosynthesis. Our study found that both salt stress alone and dopamine application downregulated the expression of Phosphoenolpyruvate (PEPC) and Chlorophyll a/b binding protein (LHCA5) genes, but dopamine treatment mitigates the down-regulation of these genes (**Figure 11**). In mesophyll cells, PEPC captures the relatively low concentration of CO_2_ from the air with high efficiency, fixing it to produce oxaloacetate and thereby completing carbon dioxide fixation (Wang et al., 2016). LHCA5 is a protein complex, its main function is to transfer light energy to Photosystem I (Knoppova et al., 2014). These results indicate that exogenous dopamine spraying can more effectively promote photosynthetic capacity in grapevines under salt stress.

Maintaining low cytoplasmic Na^+^ and Cl^-^ concentrations at the cellular level is crucial for ensuring normal metabolic activities in plants (Zhu, 2001). However, under high-salinity stress, plants often accumulate excessive Na^+^ in leaves and roots, leading to a significant decrease in K^+^ and Ca^2+^ content and consequently triggering severe ion toxicity effects (Munns and Tester, 2008). In this study, salt stress promoted the accumulation of Na^+^. However, exogenous application of dopamine effectively inhibited excessive Na^+^ accumulation, reducing the Na^+^/K^+^ ratio significantly compared to the salt alone treatment. A similar result was obtained in cotton, where exogenous melatonin treatment significantly increases K^+^ content and reduces Na^+^ accumulation in root and stem tissues (Zhu et al., 2024). The maintenance of ion homeostasis under adverse conditions is crucial for plant growth and development. *NHX1* is a Na^+^/H^+^ antiporter that expels intracellular Na^+^ to the outside of the cell under stress conditions while transporting H^+^ into the cell (Gulzar et al., 2025). Under high salt stress, the potassium transporter can transport K^+^ into the cell even under extremely low external K^+^ conditions (Yang et al., 2023). This study found that both salt stress alone and dopamine application up-regulated the expression of *NHX1* and *HKT13* genes, but exogenous dopamine application significantly up-regulated the expression of the *NHX1* gene to approximately 1.6 times that of salt treatment alone (**Figure 11**). These results indicate that under salt stress, exogenous dopamine spraying can effectively regulate ion balance in grape seedlings.

Environmental stress can trigger the accumulation of ROS in plant cells, leading to damage of cell membranes and lipid peroxidation (Sharma et al., 2012). To counteract external stress, plant cells employ their endogenous antioxidant enzyme systems to scavenge ROS and maintain dynamic balance (Huang et al., 2019). In this study, exogenous dopamine application significantly enhanced the activities of SOD, POD, CAT, GPX, and GR. Correspondingly, the O_2_^−^, H_2_O_2_, and MDA contents in grape leaves significantly decreased. These findings align with research on maize (Chen et al., 2018), where exogenous melatonin alleviated reactive oxygen species burst and protected the photosynthetic activity in maize seedlings under salt stress. These results suggested that dopamine may enhance the salt tolerance through activating the antioxidant enzyme system. GPX and GR are important members of AsA-GSH cycle. As an important substrate, GSH can scavenge free radicals generated within cells, thereby reducing cell membrane damage caused by lipid peroxidation (Ribas et al., 2014). In soybean, exogenous prohexadione-calcium enhances the yield under saline-alkali stress by modulating ascorbate-glutathione defense system (Yu et al., 2025). In apple, exogenous application of dopamine significantly increased all up-regulation of all AsA-GSH related genes compared with cadmium stress (Cao et al., 2023). In this study, KEGG pathway analysis revealed that the DEGs from NaCl vs CK and DA vs CK were primarily enriched in glutathione metabolic pathway, and approximately 90% of these GST-related DEGs showed a significant up-regulation trend. Furthermore, exogenous application of dopamine significantly up-regulated the expression of GST genes (*GSTXA*, *GSTU8*, *GSTX1*, *GSTX4)* compared with salt treatment alone (**Figure 11**). GST is an important detoxifying agent, which widely participates in plant stress responses. In tomato (Yuan et al., 2024), tea plant (Chen et al., 2025), overexpression of GST genes (*SIGSTU43*, *CsGSTU7*) significantly enhances plant resistance to stresses such as low temperature and drought. So, we speculate that the glutathione metabolism pathway also plays a crucial regulatory role in dopamine-mediated grape responses to salt stress.

Phytohormones play a pivotal regulatory role in plant response and adaptation to salt stress. In addition to well-documented stress-responsive hormones such as abscisic acid (ABA), ethylene, salicylic acid (SA), and jasmonic acid (JA), growth-related hormones are also involved in plant salt stress responses, including auxin, gibberellin (GA), cytokinins (CKs), brassinosteroids (BRs), and strigolactones (SLs) (Jing et al., 2023; Latef et al., 2021; Zhao et al., 2020). In this study, KEGG pathway analysis found the DEGs from DA vs. NaCl group were primarily enriched in the plant hormone signal transduction pathway, involving IAA, ABA, CTK, and SA related hormone signaling transduction genes. Among these, most DEGs associated with CTK, SA, and ABA hormone signaling were down-regulated, while, the majority of DEGs related to the auxin signal transduction genes were significantly up-regulated, indicating the IAA signaling pathway may be involved in dopamine-mediated salt tolerance in grape. Previous studies have shown that dopamine can inhibit IAA oxidation both *in vitro* and *in vivo* by suppressing IAA oxidase activity (Kuklin and Conger, 1995; Kulma and Szopa, 2007)). The dopamine promoted cell expansion in medium supplemented with IAA and kinetin but had no effect on cells incubated in basal medium (Protacio et al., 1992). A 2023 report demonstrated that dopamine causes hypersensitivity to auxin indole-3-acetic acid by enhancing auxin activity in *Arabidopsis* (Shull et al., 2023). Meanwhile, we found that exogenous application of dopamine significantly increased the expression levels of *GH3.6*, *IAA33*, *SAUR71*, *YUCCA2* genes compared with the salt treatment alone. IAA gene is key components of the auxin signaling pathway, while *YUCCA* is a critical enzyme in auxin biosynthesis (Rozov et al., 2013). Furthermore, studies have found that Aux/IAA proteins not only indirectly influence ABA signal transduction by regulating auxin and ABA biosynthesis/metabolic pathways but also that some members directly interact with core ABA signaling components (e.g., transcription factors *ABI3* and *ABI5*) to enhance salt tolerance by modulating ABA-responsive gene expression (Zhang et al., 2021). Therefore, we speculate that the auxin signal transduction pathway may play a key role in dopamine-mediated salt tolerance in grapevines.

## Conclusion

In summary, this study reveals how exogenous dopamine enhances salt tolerance in grapevine. Dopamine treatment alleviated growth inhibition under salt stress, improving plant height and root biomass. Physiologically, dopamine reduced electrolyte leakage, reactive oxygen and MDA levels while restoring ion homeostasis by lowering leaf Na^+^ and Na^+^/K^+^ ratios. It also boosted photosynthetic efficiency and antioxidant enzyme activities. Transcriptome analysis showed DA-regulated genes were enriched in glutathione metabolism and hormone signaling, particularly GSTs and auxin-related genes. These findings clarify DA’s role in enhancing salt stress tolerance and provide insights for its practical use in viticulture.

## Data Availability

The transcriptome has been deposited into the China National Center for Bioinformation with the code CRA027203 (https://ngdc.cncb.ac.cn/gsa/search?searchTerm=CRA027203).
