# Supplementary material for: Comparative physiological and transcriptome analyzes reveal the function of exogenous dopamine in improving the tolerance to salt stress in Vitis vinifera L
Source: Front Plant Sci. 2026 May 20;17:1823723. doi: 10.3389/fpls.2026.1823723 (PMC13229642; doi:10.3389/fpls.2026.1823723)
Supplement: Supplementary file 1 [file SupplementaryFile1.docx]

Supplementary data

Supplementary TABLE 1│Sequence of the primers in the experiment for qRT-PCR.

| Gene ID | Forward primer 5' → 3' | Reverse primer 5' → 3' |
| --- | --- | --- |
| AF369524 (*VvActin*) | CCTCAACCCCAAGGCCAACAGA | ACCATCACCAGAATCCAGCACA |
| LOC100232960 | TTTTCAGTACAGTGGTGTTTGGGTT | TGGGACAACGAGGTATTTAGGAC |
| LOC100233100 | GTATTGGCTGCGATGGCTGTT | GGGTATGGATTGGCGAGTGAG |
| LOC100233103 | ACAAGATGAGATGAGGGCAGGAATG | AAGCTGTATCAACACGACGTAAGAA |
| LOC100242618 | GCTCGCTTTTGGGCTGATTTCAT | TTCCTTCACTGCCTTCTCCTGTT |
| LOC100244496 | CGGCCACCTCATTGCCTACG | CCCCTCCTCTTCCCTTCCTT |
| LOC100244516 | GAGAACAACTTCAAGCAGCCAGGA | GGATCAGATAAAGCATCAACCCAG |
| LOC100245995 | GCCTCAGTACCCATTACTCCCACA | TCCCTTTCACCAATTCCTCTATCT |
| LOC100247682 | CTATCTTGCCTCAGCACCCTTAT | GGCCTTCTCTCGGTCCTTCTTTT |
| LOC100249626 | AAGTGAGATTTTCAGGTGTGGGAT | AGTTGCTGGACGGCGACAGTTTTG |
| LOC100252825 | CTCTTCACACCATTCTCCAACGC | ACCATCATCATCACCTCCACCAC |
| LOC100254975 | TGTGGACAGCCAAAGGAGAAGAG | ACCTGGAGTACAATGGGAGAAAA |
| LOC100255844 | GTGGTCCTCTGCTAAATCCTCTT | GCCGTCCATTCTTGATCTCCTTG |
| LOC100256048 | GGGAAGAGAGTGTTGGTAGTTGG | GGGATGGGAAAGCATTATGATTG |
| LOC100264717 | TTTTTGGAGGAGAGAAGATGGGGT | TAATGAGTGGAATTTGGATGAAGT |
| LOC100267628 | CACATTTGACGCATCATACTTTAC | TTGCTCATCTTCACCATGGACCTT |
| LOC100854260 | GAGGCCGAGCTAGAATATGGGTAC | GATCATTGGAGATGGGCTGAGAAG |

Supplementary TABLE 2│Illumina-seq output statistics of 12 samples

| sample | raw_reads | raw_bases | clean_reads | clean_bases | error_rate | Q20 | Q30 | GC_pct |
| --- | --- | --- | --- | --- | --- | --- | --- | --- |
| Water_1 | 49673244 | 7.45G | 48895576 | 7.33G | 0.01 | 98.55 | 95.63 | 46.05 |
| Water_2 | 49697252 | 7.45G | 48665282 | 7.3G | 0.01 | 98.6 | 95.79 | 45.71 |
| Water_3 | 43208288 | 6.48G | 42348816 | 6.35G | 0.01 | 98.42 | 95.26 | 46.15 |
| NaCl_1 | 46648040 | 7G | 45433922 | 6.82G | 0.01 | 98.7 | 96 | 45.34 |
| NaCl_2 | 55753754 | 8.36G | 53721678 | 8.06G | 0.01 | 98.5 | 95.45 | 45.6 |
| NaCl_3 | 50078190 | 7.51G | 49022246 | 7.35G | 0.01 | 98.47 | 95.44 | 45.25 |
| DA_1 | 44101382 | 6.62G | 42970470 | 6.45G | 0.01 | 98.52 | 95.59 | 44.59 |
| DA_2 | 49375348 | 7.41G | 48251752 | 7.24G | 0.01 | 98.51 | 95.48 | 45.39 |
| DA_3 | 47542966 | 7.13G | 46560646 | 6.98G | 0.01 | 98.42 | 95.25 | 45.7 |

Supplementary Figure 1


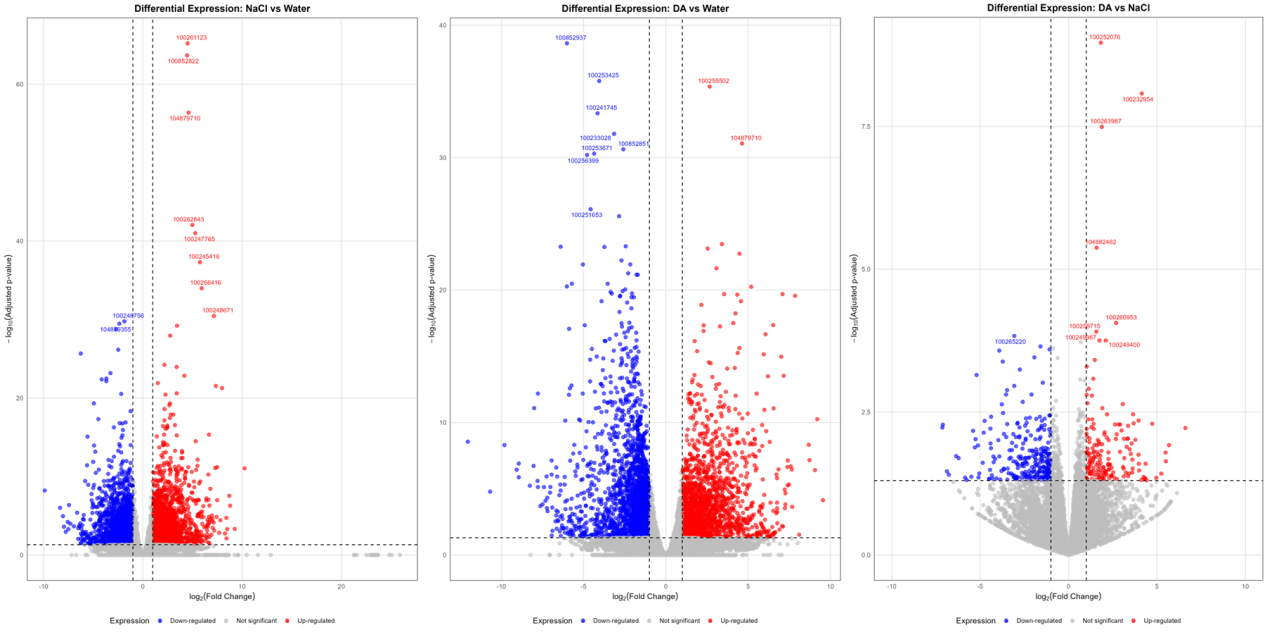


Supplementary FIGURE 1│Significance analysis of the DEGs in NaCl vs Water, Da vs Water and Da vs NaCl groups by Volcano plots.
